# Association of dietary intake of live microbes with bowel health and depression in US adults: a cross-sectional study of NHANES 2005–2010

**DOI:** 10.1265/ehpm.24-00202

**Published:** 2024-12-27

**Authors:** Jikang Shi, Qian Zhao, Zhuoshuai Liang, Heran Cui, Yawen Liu, Yi Cheng, Ming Zhang

**Affiliations:** 1 Department of Clinical Nutrition, Peking University Shenzhen Hospital, Shenzhen 518036, China; 2 Department of Preventive Medicine, School of Public Health, Suzhou Vocational Health College, Suzhou, Jiangsu, China; 3 Department of Epidemiology and Biostatistics, School of Public Health of Jilin University, Changchun 130021, China; 4 Biobank, Jilin Cancer Hospital, Changchun 130012, China; 5 The Cardiovascular Center, The First Hospital of Jilin University, Changchun 130021, Jilin, China

**Keywords:** Dietary live microbe, Bowel health, Depression, NHANES

## Abstract

**Background:**

Depression substantially impacts on quality of life, personal relationships, and self-care. Gastrointestinal disorders are the common comorbidity of depression and 24.3% of patients with depression have disordered bowel habits. Dietary intake of live microbes alters the host’s microflora and is beneficial for the prevention and control of bowel health and depression. We aim to investigate the association of dietary intake of live microbes with bowel health and depression and to further examine weather bowel health or depression mediates the therapeutic effect of live microbes.

**Methods:**

Participants’ data were obtained from the National Health and Nutrition Examination Survey (NHANES) 2005–2010, which is designed to examine the health and nutritional status of the non-institutionalized US population by a complex, multi-stage, probability sampling design. The foods were grouped into categories on the basis of estimated microbial levels: low (<10^4^ CFUs/g), medium (Med; 10^4^–10^7^ CFU/g), and high (Hi; >10^7^ CFU/g). Participants were further classified into three groups (G1: participants without MedHi foods intakes; G2: those with MedHi foods intakes greater than zero but less than the median; and G3: those with MedHi foods intakes greater than the median).

**Results:**

A total of 10,785 US adults were selected. The median of MedHi foods intake was 66.1 g/day. Participants in the G2 (OR = 0.739, 95% CI: 0.581–0.941) and G3 (OR = 0.716, 95% CI: 0.585–0.877) groups had significant association with lower risks of depression, and participants in the G3 group had significant association with lower risks of hard stools (OR = 0.885, 95% CI: 0.692–0.989) and loose stools (OR = 0.769, 95% CI: 0.585–0.954). Interestingly, further mediation analyses showed that the association of dietary live microbe intake with depression is mediated by the stool types, and the association of dietary live microbe intake with stool types is mediated by the depression (all *P* < 0.05).

**Conclusions:**

A high dietary intake of live microbes, especially a minimum of 66.1 g of MedHi foods per day, is associated with a lower risk of depression, hard stools, and loose stools consistency. Depression and bowel health mutually act as mediators in this association, indicating dietary intake of live microbes may simultaneously affect bowel health and depression.

## Graphical Abstract

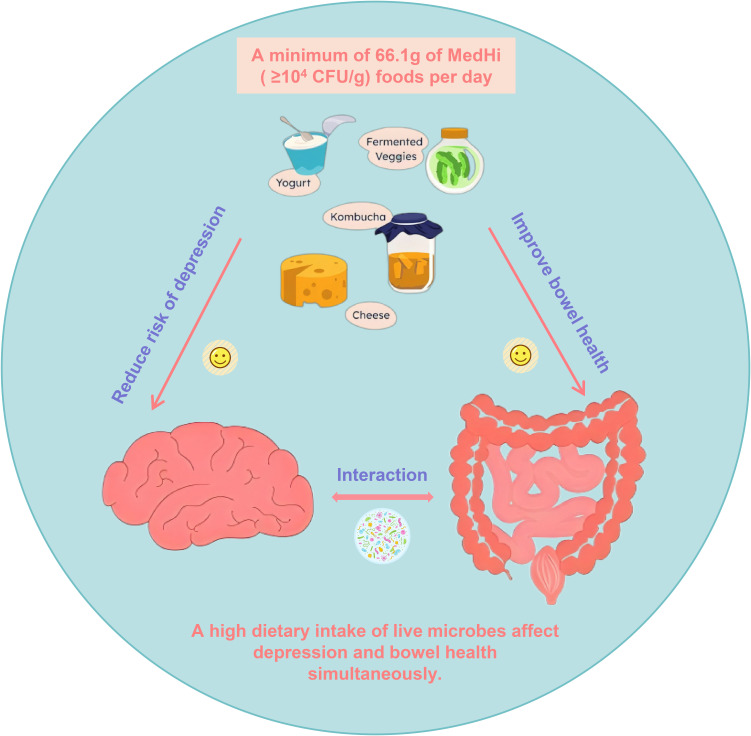

## 1. Introduction

Depression is a common mental disorder in the United States (US), with an estimated 7.8% (19.4 million) of US adults experienced at least 1 major depressive episode in 2019 [1]. Depression substantially impacts on quality of life, personal relationships, and self-care, thereby enhancing mortality, higher risk of cardiovascular events, and exacerbating of comorbid conditions [2–4]. Major depressive disorder (MDD) is a severe form of depression, with the incremental economic burden of populations with MDD increased by 37.9% from $236.6 billion to $326.2 billion between 2010 and 2018 [5].

Gastrointestinal disorders are the common comorbidity of depression [6, 7]: 24.3% of patients with depression have disordered bowel habits, 16% ones have chronic diarrhea, and 9% ones have chronic constipation [8]. Moreover, gastrointestinal side effects, associated with antidepressant treatments, such as nausea, abdominal pain, diarrhea, and constipation, are frequently observed in patients with MDD. These side effects may lead to treatment discontinuation [9]. Similarly, 15% of patients with inflammatory bowel disease (IBD) develop depression [10]. Depression may cause intestinal disturbances through the brain-gut-microbiome axis (BGMA), which constitutes a bidirectional communication system comprising neural, endocrine/metabolic, immunological and microbial signals, and vice versa [11, 12].

Dietary intake of live microbes alters the host’s microflora and is beneficial for the prevention and control of gastrointestinal diseases [13, 14], cardiovascular diseases [15–17], periodontitis [18], and depression [19, 20]. Treatment with probiotics ameliorates the intensity of depression in patients with MDD [21]. Meanwhile, probiotics is effective for acute infectious diarrhea [22], antibiotic-associated diarrhea [23] and constipation [24, 25].

Although the effect of live microbes on the treatment of depression or gastrointestinal disorders is well established, it is still unclear how dietary intake of live microbes associates with comorbidity of depression and gastrointestinal disorders. In this study, we investigated the association of the dietary intake of live microbes with the risk of depression or gastrointestinal disorders, further examining whether depression or bowel health mutually mediates the therapeutic effect of live microbes using the data from National Health and Nutrition Examination Survey (NHANES).

## 2. Materials and methods

### 2.1. Study population

NHANES is a large, ongoing, nationally representative, cross-sectional survey of the noninstitutionalized civilian population designed to monitor the nutritional, dietary, and health status of Americans. This study was approved by the National Center for Health Statistics (NCHS) and Research’s Ethics Review Committee, and each participant signed an informed consent form. Detailed descriptions of the subject recruitment, survey design, and data collection procedures are available online [26]. Participants aged 20 years and above from 2005–2010 cycles of NHANES were included in this study only if they completed the Bowel Health Questionnaire. Additionally, we excluded participants with self-reported history of inflammatory bowel disease, celiac disease, colon cancer, pregnancy and/or breastfeeding, unavailable information on live microbes or depression, and any missing data.

### 2.2. Dietary intake of live microbes

Dietary intakes were estimated using data from 24-h dietary recall collected through in-person interviews. The estimated quantities of live microbes (CFU/g) for 9388 food codes contained in 48 subgroups in the NHANES database were determined by 4 experts [27]. The foods were categorized on the basis of estimated microbial levels: low (<10^4^ CFUs/g), medium (Med, 10^4^–10^7^ CFU/g), and high (Hi, >10^7^ CFU/g). Given that the simultaneous inclusion of both Med and Hi food categories in one model indicated that their coefficients were comparable, we utilized a new category, termed ‘MedHi’, in line with the methodology of previous studies [16, 27]. MedHi referred to foods that were categorized as either having medium or high microbial content. We then calculated the grams of the new categories of food consumed for each participant, and the participants who were finally enrolled were further classified into three groups (G1: participants without MedHi foods intakes; G2: those with MedHi foods intakes greater than zero but less than the median; and G3: those with MedHi foods intakes greater than the median).

### 2.3. Bowel Health Questionnaire and Depression Questionnaires

The bowel health questionnaires from the NHANES 2005–2010 were used to analyze bowel symptoms in participants with self-reported bowel habits. Participants were shown a card with colored pictures and descriptions of the seven Bristol Stool Form Scale types (BSFS; Type 1–Type 7) and asked to ‘Please look at this card and tell me the number that corresponds with your usual or most common stool type (stool consistency)’. Stool consistency was categorized into hard stools (types 1 and 2), normal stools (types 3 and 4), and loose stools (types 5, 6 and 7) [28].

Depression Screener from the 2005–2010 NHANES was used to identify individuals with depression. This screener consisted of the Patient Health Questionnaire 9 (PHQ-9) [29]. The PHQ-9 had information about symptoms of depression on a 4 point scale (“0” = not at all; “3” = nearly every day) over the past 2 weeks with scores ranging from 0–27. A cutoff value of 10 was used to define individuals with depressive symptoms according to a previous study [30]: individuals with scores of 9 or below were classified as not exhibiting clinically significant depressive symptoms, whereas those with scores of 10 or higher were identified as having depressive symptoms of clinical relevance.

### 2.4. Covariates

General characteristics of participants included sex (male or female), race and ethnicity (Hispanic, non-Hispanic White, non-Hispanic Black, and other race), marital status (married, unmarried/cohabitation, and divorced/widowed/separated), smoking (never, former, and now), and drinking (never, former, mild [an average intake of no more than 1 drink per day for women and 2 drinks per day for men], moderate [an average intake of no more than 3 drinks per day for women and 4 drinks per day for men], and heavy [an average of 4 or more drinks per day for women and 5 or more drinks per day for men]). Body mass index (BMI) was defined as weight in kilograms divided by the square of height in meters (kg/m^2^). Body weight status in this study was classified as follows: healthy weight and underweight (BMI < 25 kg/m^2^), overweight (25.0–29.9 kg/m^2^), and obesity (BMI ≥ 30.0 kg/m^2^). Physical activity (PA) was assessed using the data of self-reported Global Physical activity Questionnaire (GPAQ) [31]. According to the physical activity guideline from the American College of Sports Medicine (ACSM), active PA was defined as 150 min of moderate PA or 75 min of vigorous PA. Except active PA, others were defined as inactive PA [32, 33].

### 2.5. Statistical analysis

Statistical analyses were performed under the complex sampling weight of NHANES according to the CDC guidelines [34]. For weighted characteristics description, continuous variables were analyzed using the survey-weighted linear regression, and categorical variables were analyzed using the survey-weighted Chi-square test. The continuous variables were presented as mean ± standard error (SE) and categorical variables were presented as percentages. Generalized linear model (GLM) was performed to analyze odds ratios (OR) and 95% confidence interval (CI) for the association of dietary intake of live microbes with depression and stool types, as well as the association of stool types with depression on the basis of Model 1 (age and sex were adjusted), and Model 2 (sex, age, race/ethnicity, marital status, smoking, drinking, BMI, physical activity, dietary fibre, and carbohydrate were adjusted). The GLM framework provided a flexible approach to model a wide range of data types by combining the linear model’s principles with a variety of probability distributions and link functions, functioning as a versatile tool in statistical analysis. All analyses were performed using the statistical software packages R (The R Foundation; version 4.2.1) and EmpowerStats (www.empowerstats.net, X&Y solutions, Inc., Boston, Massachusetts), and P-values < 0.05 was considered statistically significant.

## 3. Results

### 3.1. General characteristics of participants

A total of 10,785 participants were finally enrolled in this study, the mean age of the participants was 50.2 years. In these participants, we found status-relevant stool (8,342 participants with normal stools, 784 participants with hard stools, and 1,659 participants with loose stools) and depressive status (907 participants with depression, and 9,878 participants without depression). Moreover, the median of MedHi of total participants was 66.1 gram/day (g/d): for amount of MedHi foods consumption, participants with intakes of MedHi foods (G1: 0.0 g/d, G2: 28.4 g/d, and G3: 183.4 g/d); for depression, those with depression (28.4 g/d), and those without depression (69.9 g/d); for stool types, those with normal stools (70.3 g/d), those with hard stools (51.3 g/d), and those with loose stools (53.0 g/d). The more detailed information about general characteristics of participants are showed in Table 1.

**Table 1:** General characteristics of participants, NHANES 2005–2010 (Mean ± SE/N (weighted%))

**Characteristics**	**Total**	**Amount of MedHi foods consumption**			**Stool types**			**Depression**	
		
		**G1**	**G2**	**G3**	**Normal**	**Hard**	**Loose**	**No**	**Yes**
**Overall**	10785	3508 (32.5)	1856 (17.2)	5421 (50.3)	8342 (77.3)	784 (7.3)	1659 (15.4)	9878 (91.6)	907 (8.4)
**MedHi (median: g/day)**	66.1	0.0	28.4	183.4	70.3	51.3	53.0	69.6	28.4
**Age**	50.2 ± 17.6 (47.4)	48.8 ± 17.6 (45.6)	48.9 ± 17.7 (46.2)	51.5 ± 17.3 (48.8)	50.2 ± 17.6 (47.3)	48.5 ± 18.2 (46.2)	51.2 ± 16.7 (48.5)	50.5 ± 17.8 (47.5)	47.5 ± 15.0 (45.8)
**Sex**									
Female	5392 (51.2)	1655 (48.0)	947 (53.0)	2790 (52.5)	3932 (48.7)	547 (73.6)	913 (54.8)	4820 (50.3)	572 (62.9)
Male	5393 (48.8)	1853 (52.0)	909 (47.0)	2631 (47.5)	4410 (51.3)	237 (26.4)	746 (45.2)	5058 (49.7)	335 (37.1)
**Race/ethnicity**									
Hispanic group	2677 (11.2)	809 (11.7)	492 (11.8)	1376 (10.7)	1972 (10.5)	216 (13.5)	489 (14.2)	2422 (11.0)	255 (14.6)
Non-Hispanic White	5667 (73.9)	1618 (67.3)	985 (74.1)	3064 (77.6)	4524 (75.3)	361 (67.6)	782 (69.1)	5247 (74.6)	420 (65.3)
Non-Hispanic Black	2038 (9.9)	939 (15.4)	315 (9.4)	784 (7.0)	1538 (9.4)	184 (14.3)	316 (10.8)	1843 (9.5)	195 (15.1)
Other Race	403 (4.9)	142 (5.6)	64 (4.6)	197 (4.7)	308 (4.8)	23 (4.6)	72 (5.9)	366 (4.9)	37 (5.0)
**BMI**									
<25	3015 (30.7)	964 (29.4)	506 (30.4)	1545 (31.4)	2368 (30.9)	278 (38.5)	369 (25.5)	2796 (31.0)	219 (26.5)
25–29.9	3706 (33.7)	1141 (31.5)	647 (34.4)	1918 (34.7)	2929 (34.6)	255 (33.1)	522 (29.0)	3449 (34.1)	257 (28.5)
≥30	4064 (35.6)	1403 (39.1)	703 (35.1)	1958 (33.8)	3045 (34.5)	251 (28.4)	768 (45.5)	3633 (34.9)	431 (45.1)
**Marital status**									
Married	5915 (59.2)	1758 (54.8)	1004 (57.8)	3153 (62.2)	4607 (59.4)	383 (53.6)	925 (61.1)	5565 (60.6)	350 (41.2)
Unmarried/cohabitation	2462 (22.9)	894 (25.5)	460 (24.7)	1108 (20.8)	1914 (23.2)	202 (24.8)	346 (20.3)	2202 (22.5)	260 (27.9)
Divorced/widowed/separated	2408 (17.9)	856 (19.6)	392 (17.5)	1160 (17.1)	1821 (17.5)	199 (21.6)	388 (18.6)	2111 (17.0)	297 (30.9)
**Smoking**									
Never	5620 (52.9)	1710 (49.1)	960 (52.3)	2950 (55.1)	4343 (53.1)	467 (59.8)	810 (48.1)	5258 (53.9)	362 (38.8)
Former	2848 (25.8)	827 (22.6)	469 (23.3)	1552 (28.4)	2229 (26.0)	163 (20.0)	456 (27.2)	2658 (26.2)	190 (20.2)
Now	2317 (21.4)	971 (28.2)	427 (24.3)	919 (16.5)	1770 (20.9)	154 (20.2)	393 (24.7)	1962 (19.9)	355 (41.0)
**Drinking**									
Never	1359 (10.3)	471 (11.3)	241 (10.7)	647 (9.6)	976 (9.7)	139 (15.0)	244 (11.5)	1254 (10.3)	105 (9.9)
Former	2159 (16.6)	825 (20.1)	367 (16.8)	967 (14.5)	1627 (15.8)	172 (18.6)	360 (20.1)	1922 (16.1)	237 (23.0)
Mild	3492 (35.8)	963 (30.8)	553 (32.4)	1976 (39.8)	2780 (36.7)	231 (33.0)	481 (32.4)	3287 (36.6)	205 (25.0)
Moderate	1612 (16.7)	498 (15.4)	279 (16.7)	835 (17.5)	1295 (17.3)	100 (14.2)	217 (14.6)	1486 (16.7)	126 (16.8)
Heavy	2163 (20.6)	751 (22.4)	416 (23.4)	996 (18.7)	1664 (20.5)	142 (19.2)	357 (21.4)	1929 (20.2)	234 (25.3)
**Total-Time physical activity (min/week)**									
Inactive	5309 (46.5)	1876 (51.3)	916 (46.6)	2517 (43.8)	4025 (45.6)	432 (51.2)	852 (49.5)	4778 (45.7)	531 (58.0)
Active	5476 (53.5)	1632 (48.7)	940 (53.4)	2904 (56.2)	4317 (54.4)	352 (48.8)	807 (50.5)	5100 (54.3)	376 (42.0)

MedHi: foods containing >10^4^ CFU/g that were categorized as either having Med (10^4^–10^7^ live CFU/g) or Hi (>10^7^ live CFU/g) microbial content.

G1: those without intakes of MedHi foods

G2: those with intakes of MedHi foods (greater than zero but less than the median)

G3: those with intakes of MedHi foods (greater than or equal to the median)

### 3.2. Association of dietary intake of live microbes with stool types

Participants in the G3 group had a lower risk of hard stools than those in the G1 group (OR = 0.754, 95% CI: 0.613–0.926) after adjusting sex and age, and those in the G3 group were still of lower risks of hard stools (OR = 0.885, 95% CI: 0.692–0.989) after adjusting variables (sex, age, race/ethnicity, marital status, smoking, drinking, BMI, physical activity, dietary fibre, and carbohydrate). In addition, compared with participants in the G1 group, those in the G2 and G3 group showed significant association between dietary intake of live microbes and loose stools (for the G2 group, OR = 0.813, 95% CI: 0.696–0.950; for the G3 group, OR = 0.748, 95% CI: 0.651–0.859) after adjusting sex and age. However, there only existed significant association between dietary intake of live microbes and loose stools in the G3 group (OR = 0.769, 95% CI: 0.585–0.954) after adjusting variables (sex, age, race/ethnicity, marital status, smoking, drinking, BMI, physical activity, dietary fibre, and carbohydrate) (Table 2).

**Table 2:** Association between dietary intake of live microbes and stool types in US adult population, NHANES 2005–2010

**Food category to ** **stool types**	**Model 1**	**Model 2**
	
	**OR (95%CI)**	**OR (95%CI)**
**Food category to hard stools**		
G1	1.000 (reference)	1.000 (reference)
G2	0.847 (0.655, 1.097)	0.903 (0.775, 1.130)
G3	**0.754 (0.613, 0.926)**	**0.885 (0.692, 0.989)**
**Food category to loose stools**		
G1	1.000 (reference)	1.000 (reference)
G2	**0.813 (0.696, 0.950)**	0.843 (0.726, 1.020)
G3	**0.748 (0.651, 0.859)**	**0.769 (0.585, 0.954)**

Model 1: adjusted for sex, age.

Model 2: adjusted for sex, age, race/ethnicity, marital status, smoking, drinking, BMI, physical activity, dietary fibre, and carbohydrate.

### 3.3. Association of dietary intake of live microbes with depression

Compared with participants in the G1 group, those in the G2 and G3 group showed significant association between dietary intake of live microbes and depression (for the G2 group, OR = 0.655, 95% CI: 0.524–0.819; for the G3 group, OR = 0.555, 95% CI: 0.458–0.672) after adjusting sex and age. Moreover, there still existed significant association between dietary intake of live microbes and depression in the G2 group (OR = 0.739, 95% CI: 0.581–0.941) and in the G3 group (OR = 0.716, 95% CI: 0.585–0.877) after adjusting variables (sex, age, race/ethnicity, marital status, smoking, drinking, BMI, physical activity, dietary fibre, and carbohydrate) (Table 3).

**Table 3:** Association between dietary intake of live microbes and prevalence of depression in US adult population, NHANES 2005–2010

**Food category**	**Model 1**	**Model 2**
	
	**OR (95%CI)**	**OR (95%CI)**
G1	1.000 (reference)	1.000 (reference)
G2	**0.655 (0.524, 0.819)**	**0.739 (0.581, 0.941)**
G3	**0.555 (0.458, 0.672)**	**0.716 (0.585, 0.877)**

Model 1: adjusted for sex, age.

Model 2: adjusted for sex, age, race/ethnicity, marital status, smoking, drinking, BMI, physical activity, dietary fibre, and carbohydrate.

### 3.4. Association of stool types with depression

Participants with hard stools and loose stools had high risks of depression than those with normal stools (for hard stools to depression, OR = 1.828, 95% CI: 1.353–2.471; for loose stools to depression, OR = 2.147, 95% CI: 1.649–2.796) after adjusting variables (sex, age, race/ethnicity, marital status, smoking, drinking, BMI, physical activity, dietary fibre, and carbohydrate). Moreover, participants with depression had high risks of hard stools and loose stools than those without depression (for depression to hard stools, OR = 1.825, 95% CI: 1.352–2.462; for depression to loose stools, OR = 2.128, 95% CI: 1.633–2.773) after adjusting for all of the variables (Table 4).

**Table 4:** Association between stool types and depression in US adult population, NHANES 2005–2010

**Variables**	**Model 1**	**Model 2**
	
	**OR (95%CI)**	**OR (95%CI)**
**stool types to depression**		
Normal	1.000 (reference)	1.000 (reference)
Hard	**1.840 (1.391, 2.435)**	**1.828 (1.353, 2.471)**
Loose	**2.378 (1.826, 3.096)**	**2.147 (1.649, 2.796)**
**depression to hard stools**		
No	1.000 (reference)	1.000 (reference)
Yes	**1.853 (1.404, 2.446)**	**1.825 (1.352, 2.462)**
**depression to loose stools**		
No	1.000 (reference)	1.000 (reference)
Yes	**2.376 (1.825, 3.093)**	**2.128 (1.633, 2.773)**

Model 1: adjusted for sex, age.

Model 2: adjusted for sex, age, race/ethnicity, marital status, smoking, drinking, BMI, physical activity, dietary fibre, and carbohydrate.

### 3.5. Mediation analyses

**Fig. 1:**
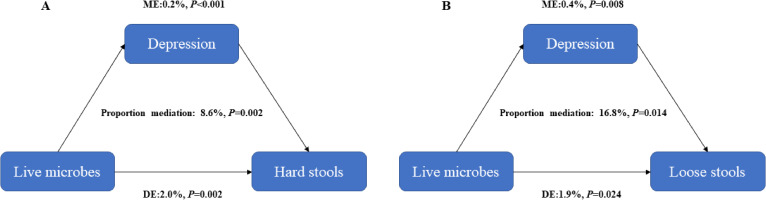
Estimated mediation effects of depression on the association between dietary live microbes and stool types (A) hard stools (B) loose stools. IE, the estimate of the indirect effect; DE, the estimate of the direct effect; Proportion mediation = IE/(DE + IE)

**Fig. 2:**
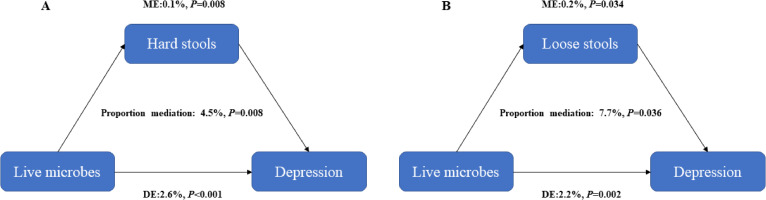
Estimated mediation effects of stool types on the association between dietary live microbes and depression (A) hard stools (B) loose stools. IE, the estimate of the indirect effect; DE, the estimate of the direct effect; Proportion mediation = IE/(DE + IE)

We further performed the mediation analyses to evaluate the potential mediation effects of depression on the association between dietary intake of live microbes and stool types, finding that depression had significant mediated effects on the association of dietary intake of live microbes with hard stools and loose stools, and the proportion of mediation was 8.6% and 16.8% respectively (all *P* < 0.05) (Fig. 1). Similarly, the mediation analyses were also performed to evaluate the potential mediation effects of stool types on the association between dietary intake of live microbes and depression. There were significant mediated effects of hard stools and loose stools on the association between dietary intake of live microbes and depression, and the proportion of mediation was 4.5% and 7.7% respectively (all *P* < 0.05) (Fig. 2).

## 4. Discussion

In this study, we unveiled that there existed lower risks of depression in the G2 and G3 group and lower risks of hard stools and loose stools in the G3 group; moreover, depression and stool types acted as mediators, and *vice versa*. Furthermore, we documented that dietary intake of live microbes was eligible for simultaneously intervening on depression and stool types.

The phenomenon of comorbidity of depression and gastrointestinal disorders was found [8, 10]. A meta-analysis fleshed out the bidirectional risk of depression and IBD (patients with depression had a 2-fold increased risk of IBD, and IBD patients had a 1.6-fold increased risk of depression) [35]. We further confirmed the bidirectional association between stool types (hard stool and loose stool) and high risk of depression in this study.

We founded that dietary intake of live microbes was required for simultaneously intervening on depression and stool types. Indeed, BGMA, a complex link between the brain and the gut, is responsible for the bidirectional risk of depression and gastrointestinal disorders: the bottom-up signaling of BGMA links gastrointestinal disorders with distorted thinking, maladaptive coping, and mood disturbance; moreover, the top-down signaling of BGMA contributes to the nexus from psychological factors to gastrointestinal disorders. Neural signal networks, immune signal networks, and chemical signal networks render the function of BGMA. Briefly, the vagus nerve [36], Th17/Treg activity [37], microglia [38], inflammatory cytokines [39], short chain fatty acids [40, 41], and gut microbial composition [42] play critical roles in communication between gastrointestinal disorders and depression.

Gut microbiota dysbiosis in patients with MDD is associated with reduced levels of brain-derived neurotrophic factor, and *Faecalibacterium* is negatively associated with the severity of depressive symptoms [43]. Fecal microbiota transplantation (FMT) ameliorates depressive-like phenotypes of mice and depressive symptom of patients with depression [44, 45]. *Bifidobacterium* breve CCFM1025 reshapes gut microbiota community, thereby reducing depression-like behavior of mice [46]. Similarly, patients with irritable bowel syndrome (IBS) had high proportions of *Bacteroides* and *Prevotella* and high levels of inflammatory markers [47]. Treatment with *Bifidobacterium lactis* improves symptoms in patients with IBS [48]. Moreover, patients with IBS received FMT reduce the IBS symptoms and the dysbiosis index [49]. Consistent with those above discoveries, we further corroborated that dietary intake of MedHi foods was negatively associated with risk of depression, hard stools, and loose stools. Importantly, we documented the strong relationship between increasing dietary intake of MedHi foods and increasing comorbidity of depression and gastrointestinal disorders. Thus, regulating gut microbiome is feasible for treating gastrointestinal disorders and depression, and a minimum of 66.1 g of MedHi foods per day is recommended.

Our findings demonstrated that the consumption of live microbes may have a dual preventive effect on both mental health and gastrointestinal health, highlighting the importance of dietary interventions in managing and preventing conditions like depression and gastrointestinal disorders. The mutual mediation effects of depression and bowel health in this association underscore the bidirectional relationship between mental and gut health, which is increasingly recognized in the field of preventive medicine [50]. Moreover, our study supports the notion that a diet rich in live microbes can serve as a preventive measure against depression and gastrointestinal disorders. This aligns with the growing body of evidence suggesting that the gut microbiome plays a critical role in modulating immune responses and maintaining overall health [51, 52]. By promoting a diet that includes MedHi foods, we can potentially improve public health outcomes and reduce the burden of these conditions.

Our study has some strengths. First, we explored the association of dietary intake of live microbes with bowel health and depression using large-scale, nationally representative data. Second, we revealed the effect of mediation on linking live microbes with bowel health or depression. There are also limitations in this study. First, recall bias in self-reported data is inherent limitation. Second, it is difficult to infer a causal relationship in this cross-sectional study design. Third, our analysis employed a classification system that did not differentiate between various types of microbes.

## 5. Conclusion

A high dietary intake of live microbes, especially a minimum of 66.1 g of MedHi foods per day, is associated with a lower risk of depression, hard stools, and loose stools consistency. Depression and bowel health mutually act as mediators in this association, indicating dietary intake of live microbes may simultaneously affect bowel health and depression.

## Abbreviations

NHANES: National Health and Nutrition Examination Survey
OR: odds ratio
CI: confidence interval
MDD: major depressive disorder
IBD: inflammatory bowel disease
BGMA: brain-gut-microbiome axis
BSFS: Bristol Stool Form Scale types
PHQ-9: Patient Health Questionnaire 9
BMI: Body mass index

## Declarations

### Data availability statement

This study used the NHANES publicly available data, https://wwwn.cdc.gov/nchs/nhanes.
